# Supplementary material for: Altered Nucleotide Insertion Mechanisms of Disease-Associated TERT Variants
Source: Genes (Basel). 2023 Jan 21;14(2):281. doi: 10.3390/genes14020281 (PMC9957172; doi:10.3390/genes14020281)
Supplement: Supplementary file 1 [file genes-14-00281-s001.zip › Supplemental Figures.pdf]

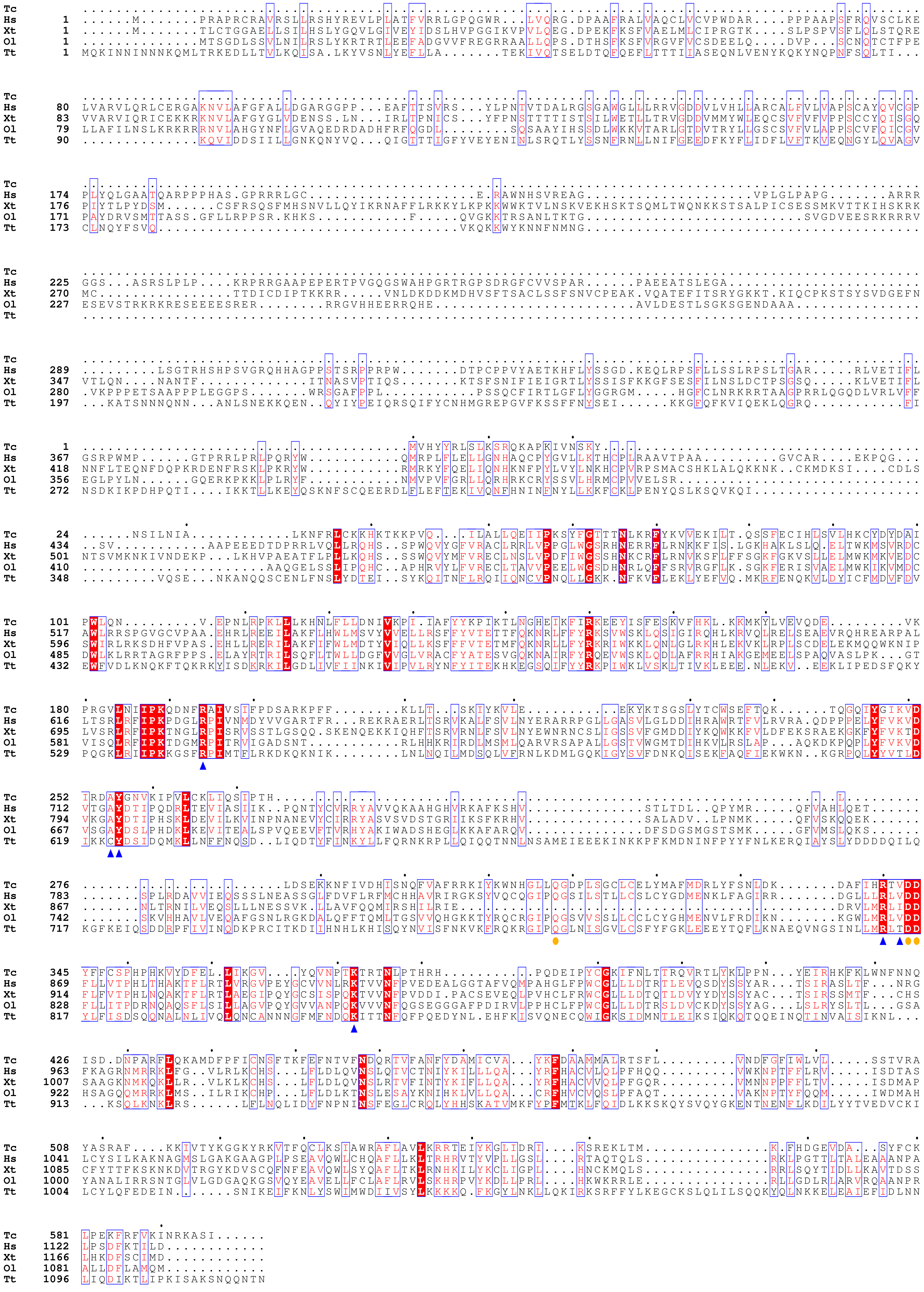

Supplemental Figure S1) Alignment of various TERT sequences . A multiple sequence alignment of TERT homologs from evolutionarily diverse organisms, (Tc: *Tribolium castaneum*, Hs: *Homo sapiens*, Xt: *Xenopus tropicalis*, Ol: *Oryzias latipes*, and Tt: *Tetrahymena thermophila*). The six residue positions investigated in this this study are indicated by the blue arrows and correspond to the tcTERT residue numbers. Additional residues discussed in the text are indicated by orange circles.

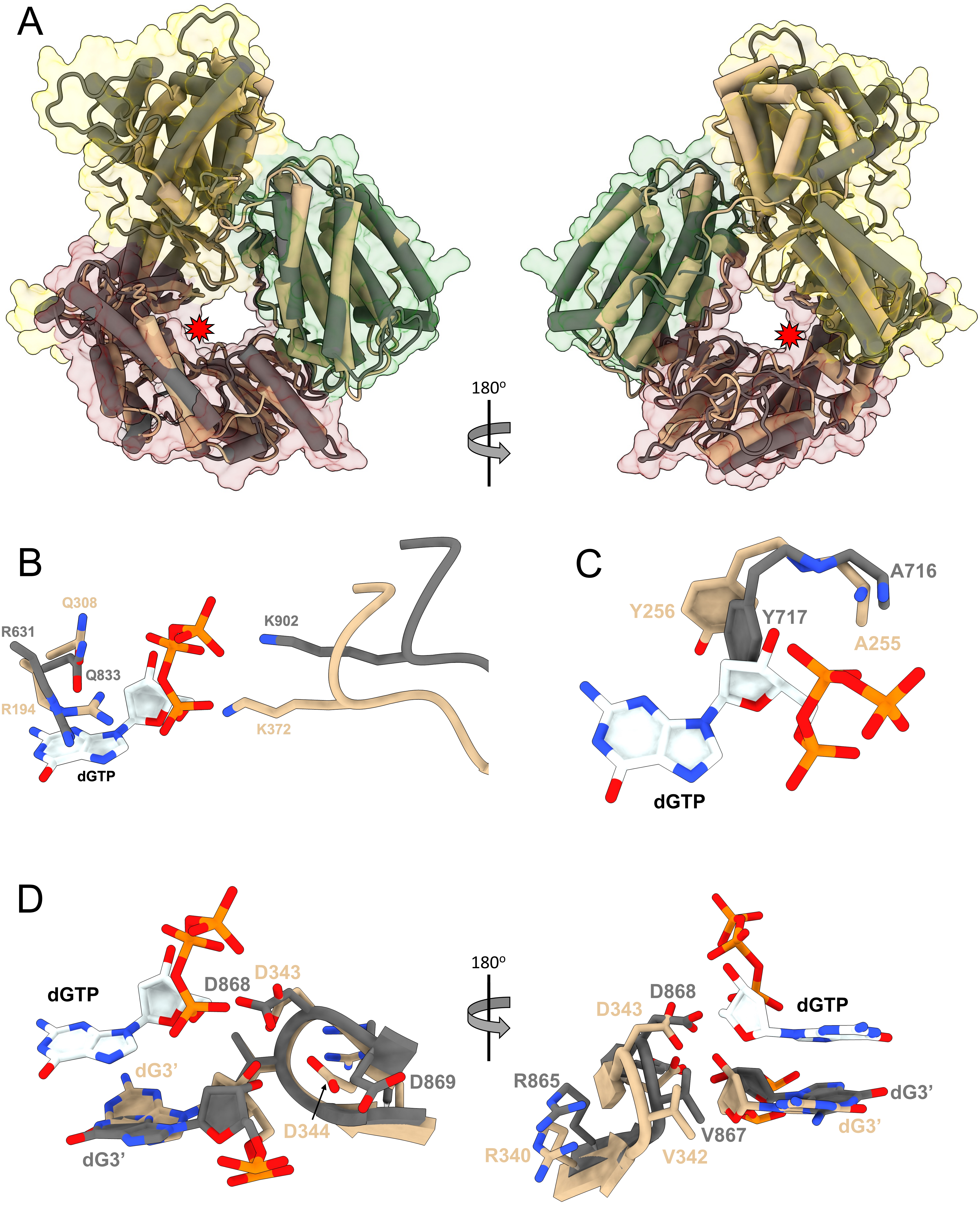

Supplemental Figure S2) Structural conservation between hTERT and tcTERT. **A**) hTERT (gray, PDB: 7QXA) and tcTERT (tan, PDB: 7KQN) are superimposed to demonstrate the conservation between the two proteins. The domains of the TERT ring are represented as surfaces with TRBD (yellow), RT (red), and CTE (green). The active site center is indicated by the red star. The TEN domain and IFD-TRAP motif of hTERT are hidden to highlight the conserved domains. (RMSD between 174 pruned atom pairs = 1.36 Å, across all 573 pairs = 12.42 Å). **B-D**) Structural conservation between tcTERT (tan) and hTERT (gray). K372 and K902 are located on a large loop which makes their absolute locations more dynamic than the other homologous residues.

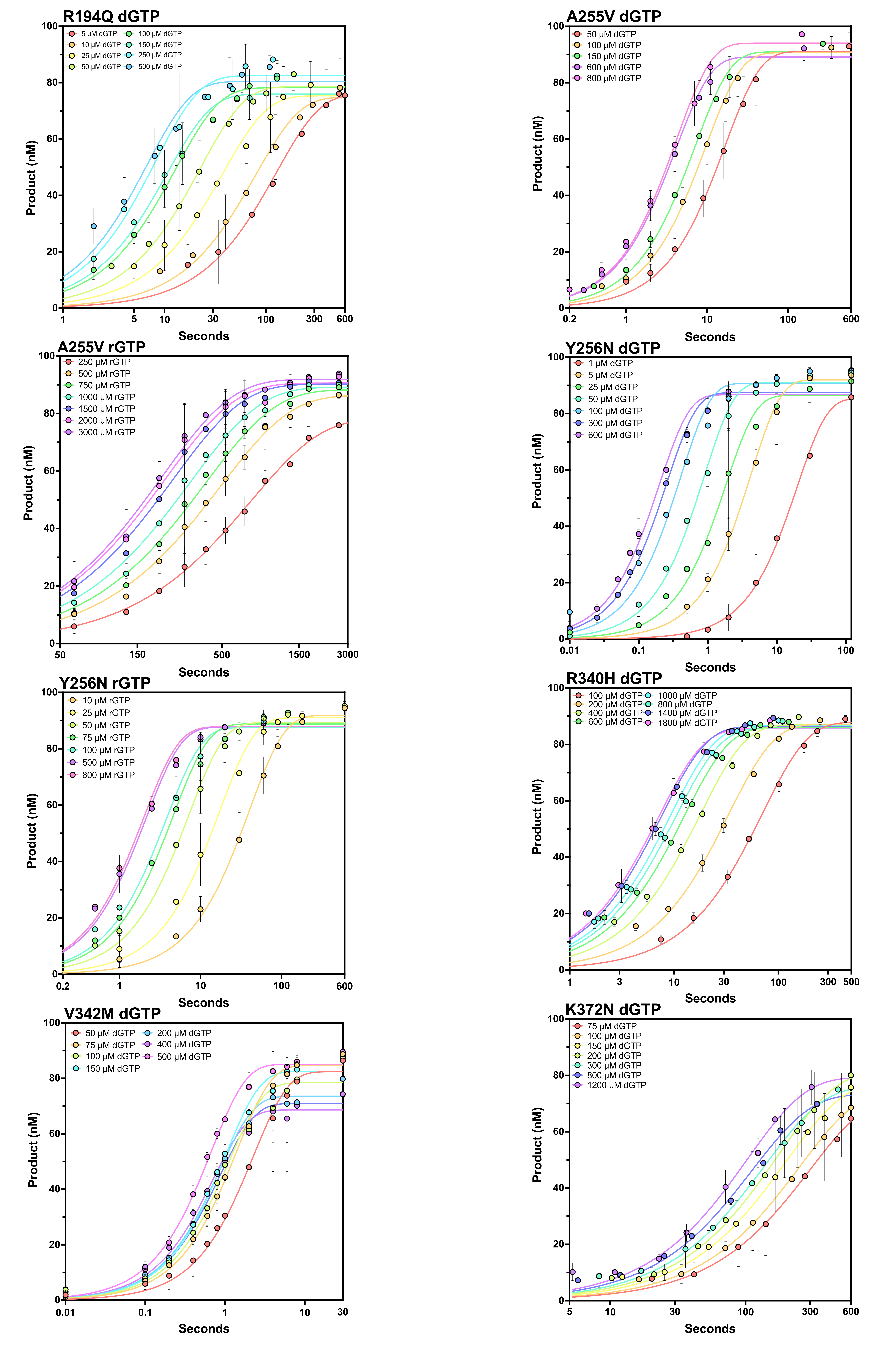

Supplemental Figure S3) Single-turnover kinetics reaction progress curves. Reaction progress curves for the specified variants were fit to Equation 1 to obtain k_{obs} and A values. The concentration of nucleotide triphosphate is indicated, and plotted values represent the mean of at least three replicates. Error bars represent the standard deviation of the mean.

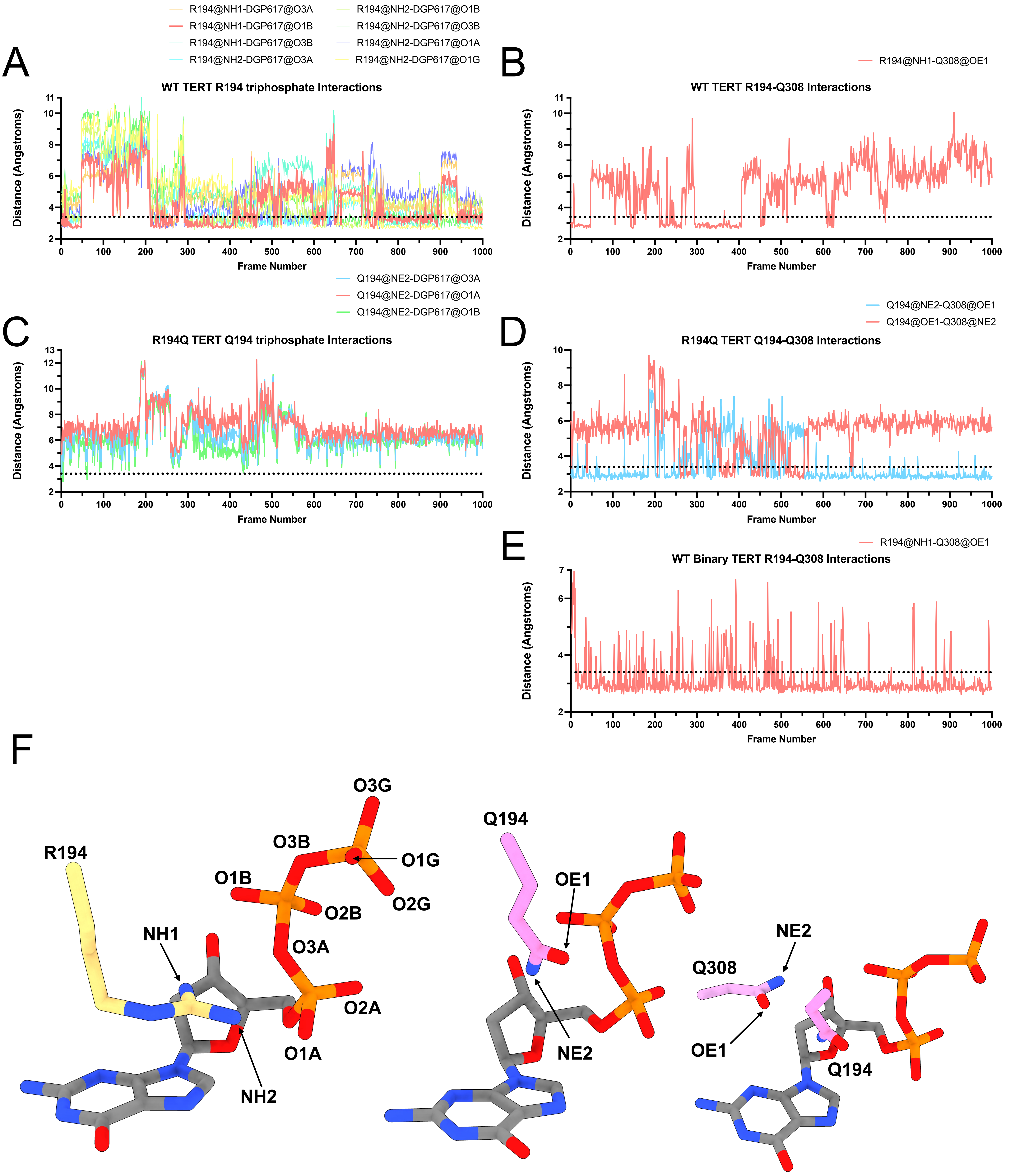

Supplemental Figure S4) Residue 194′s interactions with the incoming nucleotides triphosphate and Q308. **A**) Trajectories of R194′s interactions with the triphosphate. **B**) R194′s interactions with Q308. **C**) Trajectories of Q194′s interactions with the triphosphate. **D**) Q194′s interactions with Q308. **E**) R194′s interactions with Q308 in the WT TERT binary complex. Binary atom pair distances calculated for each trajectory are indicated and a cutoff of 3.4 Å (dotted line) was applied to determine the percent of frames with hydrogen bond interactions. **F**) Atom identities for the trajectories in panels **A-E**.
